# The Endocannabinoid System and Spermatogenesis

**DOI:** 10.3389/fendo.2013.00192

**Published:** 2013-12-16

**Authors:** Paola Grimaldi, Daniele Di Giacomo, Raffaele Geremia

**Affiliations:** ^1^Section of Anatomy, Department of Biomedicine and Prevention, University of Rome “Tor Vergata”, Rome, Italy

**Keywords:** male germ cells, spermatogenesis, endocannabinoid system, sex hormones, cannabinoid

## Abstract

Spermatogenesis is a complex process in which male germ cells undergo a mitotic phase followed by meiosis and by a morphogenetic process to form mature spermatozoa. Spermatogenesis is under the control of gonadotropins, steroid hormones and it is modulated by a complex network of autocrine and paracrine factors. These modulators ensure the correct progression of germ cell differentiation to form mature spermatozoa. Recently, it has been pointed out the relevance of endocannabinoids as critical modulators of male reproduction. Endocannabinoids are natural lipids able to bind to cannabinoid receptors and whose levels are regulated by specific biosynthetic and degradative enzymes. Together with their receptors and metabolic enzymes, they form the “endocannabinoid system” (ECS). In male reproductive tracts, they affect Sertoli cell activities, Leydig cell proliferation, germ cell differentiation, sperm motility, capacitation, and acrosome reaction. The ECS interferes with the pituitary-gonadal axis, and an intricate crosstalk between ECS and steroid hormones has been highlighted. This mini-review will focus on the involvement of the ECS in the control of spermatogenesis and on the interaction between ECS and steroid hormones.

## Introduction

Infertility affects 10–15% of couples, and it has been estimated that a male factor is responsible in approximately half of these cases. Male infertility is diagnosed with the analysis of several semen parameters, such as the number of total sperm, sperm motility, and percentage of sperm cells with a normal morphology. It is known that marijuana, the commonest recreational drug of abuse, has adverse effects on male reproductive physiology. Its use is associated with impotence, decreased testosterone plasma level, impairment of spermatogenesis, production of spermatozoa with abnormal morphology, reduction of sperm motility and viability and, more recently, with the occurrence of non-seminoma germ cell tumors (1). The identification of endogenous cannabinoids (ECBs) that mimic some effects of delta-9-THC, the active principle of *Cannabis sativa*, has opened new studies on the biological role of ECBs in male reproduction. In this mini-review we focused on the relevance of endocannabinoids and “endocannabinoid system” (ECS) in spermatogenesis and sperm functions, and on the interplay between ECS and sex hormone.

## Spermatogenesis

Spermatogenesis is a complex differentiative process starting from spermatogonial stem cells (SSCs), known as A-single (A_s_). The A_s_ cells, similarly to other stem cells, have the capability to self-renew, producing daughter A_s_ cells, and to progress into “undifferentiated spermatogonia” known as A-paired (A_pr_), and A-aligned (A_al_) that represent committed cells. The A_al_ spermatogonia then differentiate into A1-4, intermediate (In) and B spermatogonia which undergo meiosis as pre-leptotene spermatocytes (2). Spermatocytes pass sequentially through leptotene, zygotene, pachytene, and diplotene phases of prophase I, and then quickly undergo two M-phase divisions, yielding haploid spermatids, that became spermatozoa through the morphogenetic process called spermiogenesis. Sperm released from the seminiferous epithelium into the tubule lumen are still immature and are not able to fertilize an egg. Sperm maturation occur in the epididymis. During spermatogenesis, germ cells, at each stage of differentiation, are in close contact with Sertoli cells which provide physical and metabolic support for their proliferation, meiosis, and successful progression into spermatozoa. Sertoli cells proliferate quickly during perinatal period and they switch to a mature, non-proliferative state, around the onset of puberty. Since only a limited number of germ cells can be supported by each Sertoli cell (3), in adult testis, the number of Sertoli cells will be a critical factor with obvious consequences on fertility.

Spermatogenesis continues throughout life and it is regulated by a complex assortment of hormones as well as numerous locally produced factors that include growth factors, cytokines, and chemokines, that act through autocrine and paracrine pathways. Sertoli cell-secreted growth factors are known to have direct effects mainly on spermatogonia: Gdnf acts on self-renewal of SSCs and inhibits their differentiation (4), Bmp4 has both a proliferative and differentiative effect on these cells (5), and Kit Ligand (KL), acts on the kit tyrosine-kinase receptor expressed by differentiating type A spermatogonia (6) stimulating their progression into the mitotic cell cycle and reducing apoptosis (7). The major hormonal control system of spermatogenesis is the hypothalamic-pituitary-gonadal axis, based essentially on the release of two gonadotropins, luteinizing hormone (LH) and follicle stimulating hormone (FSH), under the stimulation of hypothalamic GnRHs. Leydig and Sertoli cells, the somatic cells of the testis, are primary responders to circulating gonadotropin hormones and their failure to respond appropriately, results in male infertility (8). LH stimulates Leydig cells to synthesize testosterone (T) and FSH acts on Sertoli cells stimulating their proliferation and expression of several trophic factors essential for spermatogenesis.

## The Endocannabinoid System

Endocannabinoids are lipid-signal molecules that are endogenous ligands for cannabinoid receptors, and together with enzymes responsible for their synthesis and degradation, they form the “ECS” (9). ECS is conserved from invertebrate to mammals and it assumes important role in physiological and pathological processes. The two best characterized endocannabinoids are *N*-arachidonoyl ethanolamine (AEA, anandamide) and 2 arachidonoyl glycerol (2-AG).

Endogenous cannabinoids bind to and activate their target receptors, causing several biological effects on different tissues. The main cannabinoid receptor targets type-1 (CB_1_) and type-2 (CB_2_) are seven trans-membrane G protein-coupled receptors (10). CB_1_ is widely expressed in the nervous system mainly at the terminal ends of central and peripheral neurons, but it is also expressed in ovary, uterus, testis, vas deferens, and urinary bladder. CB_2_ is mainly expressed in the cells of the immune system but it is also found in brainstem (11). ECBs are released from membrane phospholipid precursors by specific phospholipases, that are activated “on demand.” AEA synthesis is catalyzed by an *N*-acylphosphatidylethanolamine-specific phospholipase D (NAPE-PLD) (12). Similarly, the formation of 2-AG involves a rapid hydrolysis of inositol phospholipids by a specific phospholipase C (PLC) to generate diacylglycerol (DAG), which is then converted into 2-AG by an sn-1-DAG lipase (DAGL) (13). As lipid molecules, ECBs diffuse passively through the membrane, but the presence of a membrane transporter, EMT, that acts by a facilitated diffusion mechanism, has been hypothesized (14, 15). More recently an anandamide transporter named FLAT, which facilitates its translocation into cells has been identified in neural cells (16). The biological effects of ECBs depend on their lifespan in the extracellular space, which is limited by a re-uptake by cells. Once inside the cells ECBs are hydrolyzed by two specific enzymes: the fatty acid amide hydrolase (FAAH) cleaves AEA into arachidonic acid and ethanolamine, and the monoacylglycerol lipase (MAGL) (17) transforms the 2-AG into arachidonic acid and glycerol (18).

AEA, but not 2-AG, behaves also as an endovanilloid binding to the type-1 vanilloid receptor (transient receptor potential vanilloid 1, TRPV1) at an intracellular site (19). TRPV1 is a six trans-membrane spanning non-selective cation channel, whose expression is found mainly in specialized sensory neurons that detect painful stimuli (20). However it is now established that TRPV1 is expressed also in non-neuronal cells, such as keratinocytes and epithelial and endothelial cells, where it could play a wide variety of physiological functions.

## The Endocannabinoid System and Spermatogenesis

### ECS and germ cells

Following the discovery of ECS, many studies about its expression and function in male reproductive system have been carried out (21). The presence of components of ECS has been demonstrated in the testis, in the reproductive fluids and tracts, in different organisms from invertebrates to mammals. All the components of the ECS have been identified in mammalian germ cells, from spermatogonia to spermatozoa.

First evidence of an effect of cannabinoid in male reproduction comes from a study in sea urchin in which it was demonstrated that exogenous cannabinoid THC directly reduced the fertilizing capacity of sperm (22) through the inhibition of the acrosome reaction (23). Next, endogenous cannabinoid AEA was shown to induce the same effects of THC on sea urchin sperm (24).

Endocannabinoids have been identified in human seminal plasma (25), in the amphibian cloacal fluid (26) and in mouse epididymis (27) indicating a role in the control of sperm functions. Most of the *in vitro* studies reported an adverse effect of AEA on sperm function with inhibition of motility, capacitation and acrosome reaction, and indicated a pivotal role of CB_1_ receptor in mediating AEA effects. In humans, AEA inhibits sperm motility by decreasing mitochondrial activity and this effect was blocked by the CB_1_ receptor antagonist SR141716 (28). In boar (*Sus scropha*), a stable AEA analog, methanandamide, reduces sperm capacitation and inhibits acrosome reaction (29). Also in frog *Rana esculenta* AEA has been shown to inhibit sperm motility through CB_1_ receptor (26).

It has been described a role of CB_1_ in spermiogenesis, when elongated spermatids are remodeled to form mature spermatozoa with a change in the chromatin structure. Indeed, genetic inactivation of CB_1_ causes an inefficient histone displacement, poor chromatin condensation, and DNA damage in sperm (30), indicating a role of ECS in spermatid differentiation.

Further interesting findings supporting a role of AEA and CB_1_ receptor on sperm function arise from the gene knockout animal models. In the absence of CB_1_ signaling, sperm acquire motility precociously and the percentage of motile spermatozoa recovered from the caput of epididymis was higher with respect to wild-type mice, suggesting a physiological inhibitory regulation of endocannabinoids on sperm motility in the epididymis (31). Genetic loss of FAAH results in increased levels of AEA in the reproductive system and impairment of sperm fertilizing ability (32). These results lead to hypothesize that an “adequate tone” of AEA and the expression of CB_1_ receptor are critical in the formation of morphologically and functionally normal sperm. In support of this observation, it has been recently reported that, in rats, *in vivo* administration of HU210, a synthetic analog of THC and a potent agonist of CB receptors, causes a marked impairment of spermatogenesis with reduction in total sperm count and motility, and a deregulation of the ECS, confirming the *in vitro* observations and indicating that the use of exo-cannabinoids may influence adversely male fertility (33).

Another molecular target of AEA is the vanilloid receptor TRPV1 (34), expressed in sperm cells of mouse (35), boar (29), bull (36), and humans (37). Activation of TRPV1 receptor by AEA, seems to play a role in the stabilization of the plasma membranes in capacitated boar sperm, preventing spontaneous acrosome reaction (29). Therefore, AEA can bring different signals in sperm cells, depending on the target receptor (CB_1_ or TRPV1) that is activated.

**Figure 1:**
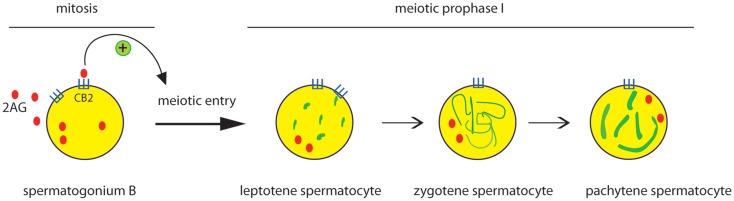
**Effect of CB_2_ activation on the early steps of spermatogenic differentiation**. Mitotic male germ cells express CB_2_ receptor and high level of 2-AG. Activation of CB_2_ receptor by 2-AG, through an autocrine pathway, promotes meiotic entry and progression of spermatogonia, as revealed by the meiotic organization of nuclear SCP3 (green) during the prophase I phases (leptotene, zygotene, pachytene).

Besides AEA, also the endocannabinoid 2-AG has been reported to affect male reproduction. Using mouse male germ cell populations at different stage of differentiation we highlighted a pivotal role of 2-AG and CB_2_ receptor in mouse spermatogenesis (35). We demonstrated that mammalian male germ cells, from mitotic to haploid stage, have a complete ECS which is modulated during spermatogenesis. Spermatogonia possess higher level of 2-AG that decreases in spermatocytes and drastically drops in spermatids. This correlates to higher level of biosynthetic (DAGL) and lower level of degrading enzymes (MAGL) in spermatogonia with respect to spermatocytes and spermatids. On the contrary, AEA levels remain unchanged during spermatogenesis and probably are crucial to maintain, locally, an appropriate “anandamide tone” for a correct progression of spermatogenesis as seen for normal development of mouse embryos (38). Interestingly, activation of CB_2_ receptor in spermatogonia promotes their progression into meiosis as revealed by an increased number of cells positive for the meiotic marker SCP3 and by the expression of premeiotic and early meiotic genes. Thus, during spermatogenesis an autocrine endocannabinoid regulation of mitotic germ cell differentiation might occur as proposed in Figure 1.

Endocannabinoid 2-AG has been also found to play a role in regulating the ability of spermatozoa to become motile during their transit in the epididymis. 2-AG levels are high in mouse spermatozoa isolated from the caput of the epididymis, where they do not move regularly, and decrease dramatically in spermatozoa isolated from the cauda, where they acquire vigorous motility, suggesting that, along the epididymis, the decrease of 2-AG levels from caput to cauda promotes start-up of spermatozoa (27). Finally mouse sperm capacitation has been found to be linked to an enhancement of the endogenous tone of both AEA and 2-AG (39), underlying the important role of ECS in regulating important step of spermatogenesis and sperm functions.

With the aim to investigate, in humans, a possible relationship between male reproductive dysfunction and deregulation of the ECS, recent studies have shown a marked reduction of AEA and 2-AG content in the seminal plasma of infertile patients. This reduction in sperm from infertile versus fertile men can be determined by either an increased ratio of degradation/biosynthesis, or by lower levels of CB_1_ mRNA expression (40, 41), indicating that the ECBs signaling is involved in the preservation of normal human sperm function.

### ECS and testicular somatic cells

Endocannabinoid system components are expressed also in somatic cells of mammalian testis. Sertoli cells possess the biochemical machinery to synthesize, transport, degrade, and bind both AEA (42) and 2-AG (43). Mouse Sertoli cells express a functional CB_2_ receptor, an AEA membrane transporter and the AEA-degrading enzyme FAAH (42). AEA has been shown to have a pro-apoptotic effect on Sertoli cells, inducing DNA fragmentation. Lower level of AEA correlates with higher level of FAAH protein and with a decrease in Sertoli cell apoptosis, suggesting a protective and pro-survival role of FAAH in Sertoli cells. More interestingly, FAAH activity and expression is hormonally up-regulated in Sertoli cells by FSH and estrogen (43, 44).

Rat Leydig cells express CB_1_ which is modulated during development and it negatively correlates to cell division. Immature Leydig cells in mitosis were negative for CB_1_, while immature non-mitotic Leydig cells were positive, indicating a negative effect of CB_1_ on Leydig cell proliferation and suggesting that their differentiation may depend on the ECS (45).

## The Endocannabinoid System and Sex Hormone

As described above, the ECS is widely distributed in testicular cells and it is an important regulator of spermatogenesis and sperm functions. Recently, many evidence indicate the existence of interplay between ECS and sex hormones, testosterone and estrogen, thus stressing the relevant role of ECS in regulating male reproduction. Testosterone is produced by Leydig cells under the stimulation of LH and it is essential for the occurrence of events like blood-testis-barrier formation, germ cells progression beyond meiosis, mature sperm release. Sertoli cells are the major cellular target for the testosterone signaling and the absence of testosterone or of the androgen receptor, results in the failure of spermatogenesis and infertility. Several studies on human males smoking cannabis, reported a decrease in plasma levels of testosterone, FSH, and LH and this effect was also evident in animal studies after acute and chronic administration of THC (46, 47). Decreased levels of testosterone correlate to an inhibitory effect of cannabinoids on male sexual behavior (48). Moreover *in vitro* studies on Leydig cells showed a decrease in testosterone secretion induced by THC (49). Similarly endogenous cannabinoid AEA suppresses LH and testosterone levels in wild-type, but not in CB_1_ knockout mice (50), providing evidence that the ECS acts to suppress testosterone levels.

It is now well documented that, beside testosterone, also estrogens are important modulator of male reproduction (51). The presence of estrogens in male reproductive tracts of numerous mammals has been reported (52). Aromatase is the enzyme that converts irreversibly androgen into estrogens and is expressed, in mammals, in all testicular cells except peritubular cells. The biological effects of estrogens are mediated by the estrogen receptors α (ERα) and β (ERβ), both expressed in mammalian testis. A role of estrogens in spermatogenesis is strongly supported by the observation that mice lacking estrogen receptors or aromatase are infertile and show impaired spermatogenesis in adulthood (53, 54).

Between all the components of ECS, the AEA-degrading enzyme *faah* gene has been demonstrated to be the only gene to be hormonally regulated in the testis. In Sertoli cells, FSH regulates FAAH expression and activity by triggering protein kinase A or aromatase-dependent pathway (42).

The PKA-dependent pathway enhances FAAH activity by inducing phosphorylation of other proteins that could activate the enzyme. On the other hand, the aromatase-dependent pathway, that leads to the conversion of testosterone into estrogens, induces FAAH expression at transcriptional level. Indeed we recently clarify the molecular mechanisms by which estrogens directly up-regulate *faah* gene transcription (55). This involves direct binding of ER to the ERE sites in the *faah* promoter and the induction of epigenetic modifications in order to confer transcriptional competence.

**Figure 2:**
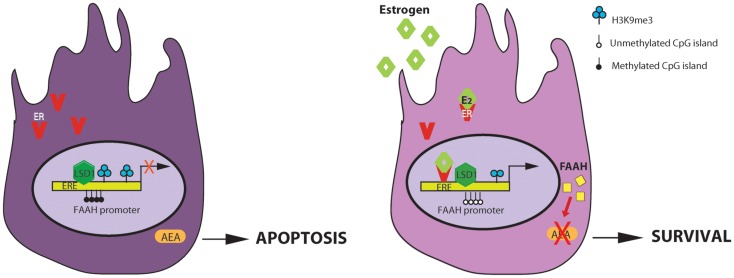
**Regulation of AEA-degrading enzyme FAAH expression by estrogen in Sertoli cells**. E_2_ regulates FAAH transcription by direct binding of estrogen receptor (ER) and epigenetic mechanisms including histone modification and DNA methylation. On the left: in the absence of estrogens, faah proximal promoter is methylated at DNA CpG sites and at lysine 9 of H3 histone and it is not competent for transcription. The final outcome is an increase AEA-induced apoptosis of Sertoli cells. On the right: estrogens activates the AEA-degrading enzyme FAAH transcription, through ER binding at ERE sites and reduction of DNA and H3K9me3 methylation. The direct/indirect interaction with histone demethylase LSD1, constitutively recruited at this site, is necessary for estrogen-induced transcription. The final outcome is a decrease of AEA-induced apoptosis of Sertoli cells (ERE, estrogen response element).

As presented in Figure 2, in Sertoli cells, E_2_ engages ER, which binds to ERE sites in the *faah* proximal promoter determining demethylation of both DNA, at CpG site, and histone H3, at lysine 9 (H3K9). The presence of histone demethylase LSD1, which is recruited at this site, ensures estrogens stimulation of *faah* transcription. LSD1 could interact with ligand-bound ER or with other different partners and activate gene transcription. The biological relevance of E_2_-stimulation of FAAH expression consists in decreasing AEA levels in Sertoli cells and protect them against apoptosis induced by AEA. The pro-survival role of E_2_ in Sertoli cells has a clear impact on spermatogenesis. In fact regulation of Sertoli cell apoptosis could be important to maintain their population size, and consequently, to sustain a normal spermatogenic output.

This is not the only example about the cross-talks between estrogens and ECS in the testis. Recent evidences reveal that estrogens affect spermiogenesis and regulate chromatin remodeling of germ cells (56). Indeed, in mice, genetic loss of CB_1_ receptor causes a reduction in FSH and estrogen plasma levels and alteration in spermatid differentiation due to an inefficient histone displacement in the sperm. Estrogens treatment is able to rescue histone displacement suggesting a role in preserving chromatin condensation in spermatozoa.

## Concluding Remarks

In this mini-review we highlighted the physiological role of ECS and its interplay with sex hormones, in male reproduction. A full comprehension of the molecular events regulated by ECS in the testis will allow to better define the “protective” role of this system in maintaining and ensuring the correct progression of spermatogenesis and the formation of mature and fertilizing sperm. Interfering with this system by exposure to exogenous cannabinoids, may alter the physiological function of ECS in male reproduction thus affecting male fertility.

## Conflict of Interest Statement

The authors declare that the research was conducted in the absence of any commercial or financial relationships that could be construed as a potential conflict of interest.
